# Combined BRD4 and CDK9 inhibition as a new therapeutic approach in malignant rhabdoid tumors

**DOI:** 10.18632/oncotarget.18583

**Published:** 2017-06-21

**Authors:** Natalia Moreno, Till Holsten, Julius Mertins, Annabelle Zhogbi, Pascal Johann, Marcel Kool, Michael Meisterernst, Kornelius Kerl

**Affiliations:** ^1^ Institute of Molecular Tumor Biology, Westfalian Wilhelms University, Muenster, Germany; ^2^ University Children’s Hospital Muenster, Department of Pediatric Hematology and Oncology, Muenster, Germany; ^3^ Division of Pediatric Neurooncology, German Cancer Research Center (DKFZ), Heidelberg, Germany

**Keywords:** CDK9, BRD4, rhabdoid tumors, synergistic, SMARCB1

## Abstract

Rhabdoid tumors are caused by the deletion of *SMARCB1*, whose protein encodes the SMARCB1 subunit of the chromatin remodeling complex SWI/SNF that is involved in global chromatin organization and gene expression control. Simultaneously inhibiting the main players involved in the deregulated transcription machinery is a promising option for preventing exaggerated tumor cell proliferation and survival as it may bypass compensatory mechanisms. In support of this hypothesis, we report efficient impairment of cellular proliferation and strong induction of cell death elicited by inhibition of bromodomain protein BRD4 and transcription kinase CDK9 using small molecular compounds. Combination of both compounds efficiently represses antiapoptotic genes and the oncogene *MYC*. Our results provide a novel approach for the treatment of RT.

## INTRODUCTION

Rhabdoid tumors (RT) are rare and highly aggressive malignancies occurring predominantly in infants and young children. These neoplasms arise in multiple tissues, including kidneys (RTK = rhabdoid tumor of the kidney), central nervous system (AT/RT = atypical teratoid/rhabdoid tumor) and soft tissue (MRT = malignant rhabdoid tumor) [1, 2]. Prognosis has improved significantly over the last years, but as overall survival remains poor [3], new therapeutic approaches are urgently required.

RT are genetically characterized by biallelic loss of *SMARCB1* (also known as *hSNF*, *INI1* or *BAF47*). SMARCB1 is a core component of the ATP-dependent SWI/SNF complex [4], which alters gene expression by remodeling chromatin accessibility for the transcription machinery [5–7]. Loss of SMARCB1 leads to dysregulation of multiple signaling cascades, including the Sonic-hedgehog [8], WNT [9] and MYC pathways [10], and also affects cyclin dependent kinases [11]. Nearly all RT harbour loss of *SMARCB1;* in rare cases the *SMARCA4* [12] gene is lost, which codes for the BRG1 ATPase. As RT lack other recurrent mutations, epigenetic events likely play a crucial role in tumorigenesis of these malignancies [6, 10]. As such, RT represent a unique and tractable model that could lead to insights for other tumor entities that demonstrate a more complicated mutational background [13–15].

RT tumorigenesis involves bromodomain transcriptional regulation [8, 10, 16]. Bromodomains act as key elements of transcriptional control and of mitotic activity [17, 18]. BRD4, a prevalent member of the human BET (bromodomain and extraterminal) protein family binds acetylated histones during mitosis to maintain chromatin structure in the daughter cell [19, 20]. Simultaneously, it mediates accessibility of the transcription machinery to specific chromatin regions, ensuring early re-initiation of transcription following mitosis [21]. During transcription pausing, BRD4 recruits the positive transcription elongation factor b (P-TEFb), which phosphorylates the RNA Pol II C-terminal domain and promotes transcription elongation [22, 23]. However, the kinase part of P-TEFb, CDK9, does not only phosphorylate RNA Pol II, but also the DRB sensitivity-inducing factor (DSIF) and negative elongation factor (NELF), which then dissociate from the polymerase [24]. The complex molecular interactions, finally, allow initiation of productive RNA synthesis [25]. Molecules that are involved in these processes are of particular interest in view of identifying targets for chemotherapeutic approaches. BRD4, of note, is known to act as a key player in Sonic-hedgehog signaling that again is a driver of RT tumorigenesis [8, 10, 16, 26]. Hence, inhibition of BRD4 as well as CDK9 with small molecule inhibitors have been demonstrated to display antitumoral effects in various studies [27, 28]. As RT are characterized by altered functionality of the SWI/SNF chromatin remodeling complex, the entity represents a promising option for testing novel inhibitor compounds with an impact on specific molecules involved in epigenetic and transcriptional regulation mechanisms.

In this study, we demonstrate anti-proliferative effects and induction of apoptosis by a combined treatment with BRD4- and CDK9 inhibitors in malignant rhabdoid tumors.

## RESULTS

### Simultaneous inhibition of BRD4 and CDK9 impairs RT growth *in vitro* and *in vivo*

BRD4 is involved in transcription regulation by recruiting P-TEFb and is inhibited by compounds like iBET and JQ1. CDK9, the kinase of P-TEFb, promotes transcription elongation [29] by phosphorylation of RNAPII and is blocked by small molecular compounds like DRB [30] or LDC067. As RT have defects in the chromatin remodeling complex SWI/SNF that lead to general transcription deregulation, we evaluated whether targeting transcription control by these two different mechanisms, namely inhibition of BRD4 and CDK9, is a promising approach for treatment of these tumors. We used two different CDK9i (DRB and LDC067) and two BRD4i (JQ1 and iBET) in five RT cell lines (G401, BT16, KD, A204, MON) to assess if these inhibitors cooperate on blocking tumor cell growth and on induction of apoptosis. Both combinations of BRD4i and CDK9i (DRB plus iBET and LDC067 plus JQ1) had synergistic or additive effects on inhibiting cell proliferation in MTT assays (Supplementary Figure 1A and 1B).

**Figure 1:**
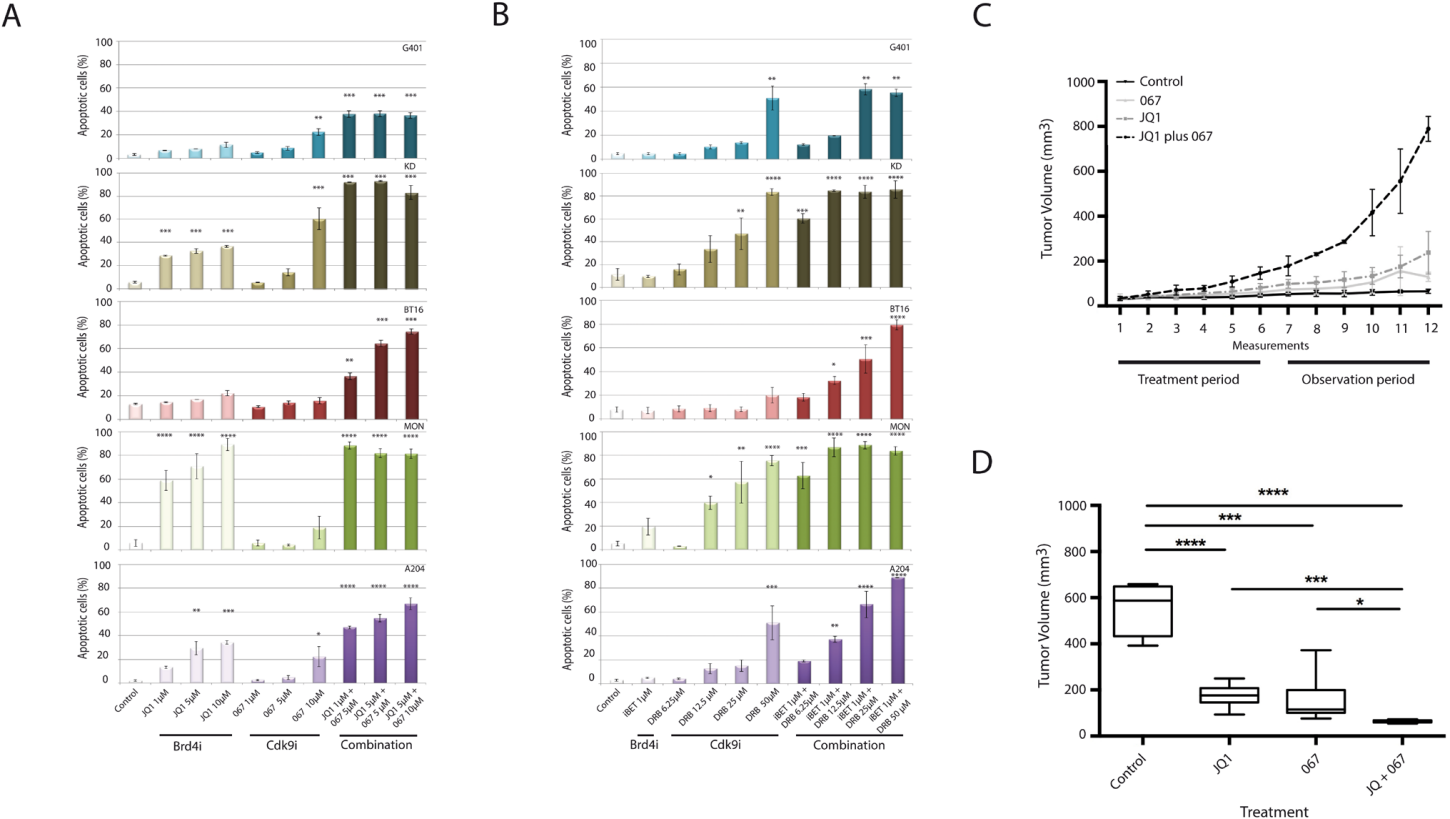
Simultaneous inhibition of BRD4 and CDK9 synergistically induces apoptosis in rhabdoid tumor cells *in vitro* and inhibits cell proliferation *in vivo* The indicated RT cell lines were treated with BRD4 and CDK9 inhibitors as single compounds or as a combination of both at different concentrations. Evaluation of apoptosis by flow cytometry shows a strong synergism on apoptosis induction after combined treatments with JQ1/LDC067 **(A)** or iBET/DRB **(B)**. Rhabdoid tumor xenograft mice were treated with Vehicle, JQ1, LDC067 or simultaneously with JQ1 plus LDC067 during 3 weeks, followed by an observation period of three additional weeks. Tumor size was measured twice a week by digital caliper **(C)**. Box-plots show the average tumor volume at the end of the experiment **(D)**. *p<0.05, **p<0.01, ***p<0.001, ****p < 0.0001 (ANOVA One-way test).

In order to examine whether the observed antiproliferative effect was accompanied by cell death, apoptosis was measured by flow cytometry in all obtainable RT cell lines (G401, BT16, KD, MON and A204). Single treatment of these cell lines with either BRD4i (iBET or JQ1) or CDK9i (DRB, LDC067) induced apoptosis in a concentration-dependent manner (Figure 1A and 1B). In support of our proliferation assay data, the combined application of these inhibitors induced high levels of apoptosis even at very low concentrations (Figure 1A and 1B). While the effect of combinatorial treatment could be principally observed in every single cell line, the absolute extent of cells undergoing apoptosis varied within different cell lines with KD and MON cells showing the highest sensitivity for treatment with both BRD4 and CDK9 inhibitors. As expected, use of the less specific CDK inhibitor DRB generally resulted in slightly higher amount of apoptotic cells than treatment with the highly specific CDk9-Inhibitor LDC067.

To further investigate the growth inhibitory mechanisms of BRD4i and CDK9i, cell cycle effects were studied using flow cytometry. BRD4i as well as CDK9i individually increased as single compounds the fraction of cells in G1 phase in a concentration-dependent manner (Supplementary Figure 1C and 1D) which is consistent with previously published data [31, 32].

Based on the synergism of BRD4i and CDK9i on induction of apoptosis, as a next step towards a therapeutic strategy, xenograft models were used by injecting G401-RT cells subcutaneously into the flank of NOD/SCID mice. Once tumor volumes reached 50-80 mm^3^ mice were randomized into four groups: I.) Vehicle; II.) JQ1; III.) LDC067 or IV.) JQ1/ LDC067.

None of the treatments elicited toxicity in these animals documented by no weight loss and good general conditions during the experiment. Xenograft mice receiving single treatments of JQ1 or LDC067 showed a reduction of tumor volume compared to vehicle-treated mice. Nevertheless, the combined application of CDK9i and BRD4i promoted a more effective suppression of tumor growth (p<0.0001) (Figure 1C and 1D).

Together, these data demonstrate that simultaneous inhibition of CDK9 and BRD4 acts synergistically on suppressing tumor proliferation.

### BRD4i and CDK9i synergistically down-regulate anti-apoptotic genes

**Figure 2:**
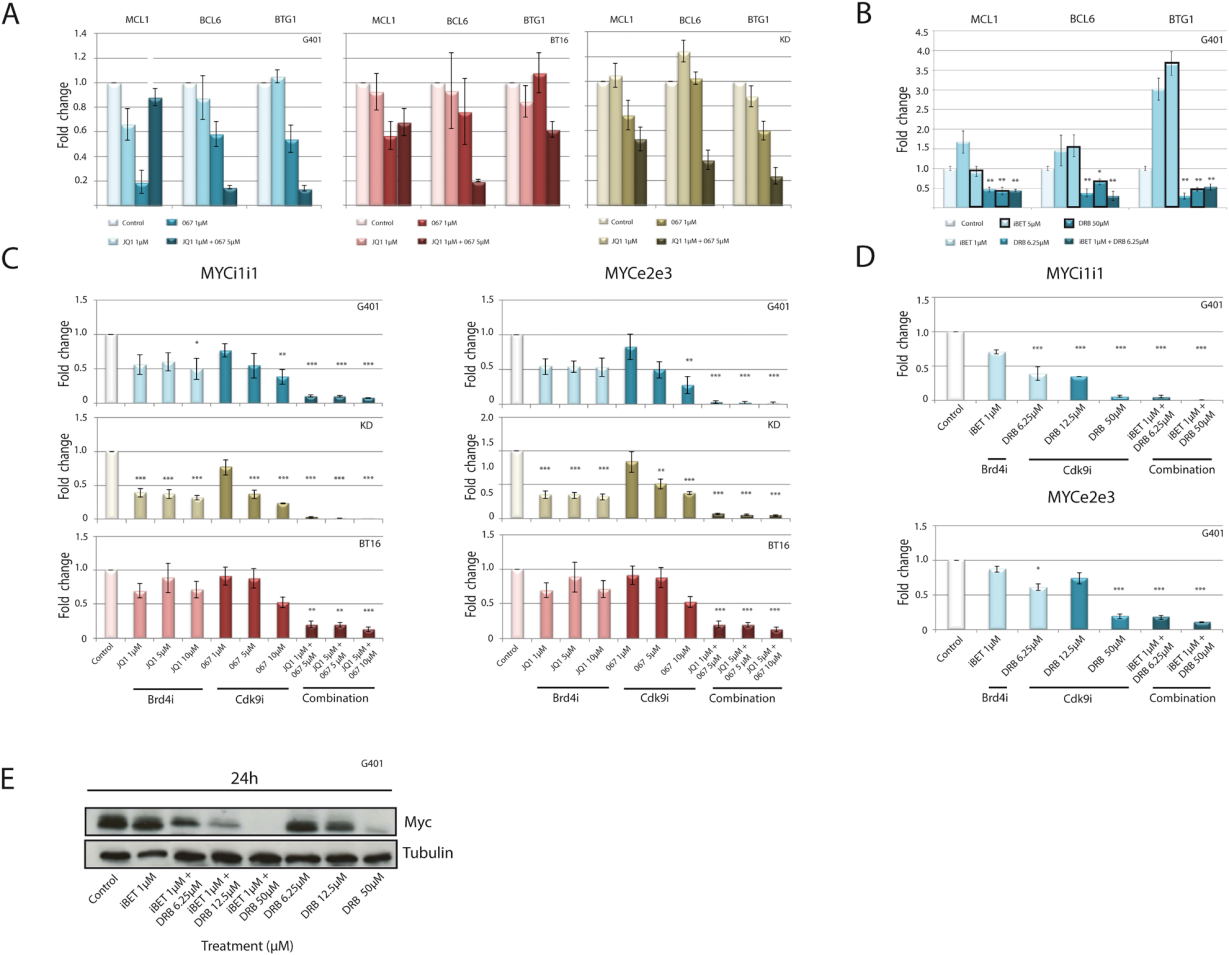
Down-regulation of anti-apoptotic genes and MYC expression in response to combined CDK9 and BRD4 inhibition Rhabdoid tumor cell lines were treated for 120 min with increasing concentrations of BRD4 or CDK9 inhibitors alone or in combination. Expression of anti-apoptotic genes was analyzed by RT-qPCR. A significant down-regulation of anti-apoptotic genes is evident after exposure to JQ1/ LDC067 **(A)** or iBET/DRB **(B)**. RT-qPCR analysis of *de novo transcription* was performed to assess if simultaneous treatment of RT cell lines can affect newly synthesized MYC mRNA using exon-exon primers (MYCe2e3) or intron-intron primers (MYCi1i1) after treatment with JQ1/ LDC067 **(C)** or iBET/DRB **(D)**. Despite repressed MYC mRNA production, protein levels remain stable 24h after treatment of G401 cells with iBET or with DRB at low doses. Combination of both compounds synergistically reduces MYC protein levels **(E)**. *p<0.05, **p < 0.01, ***p < 0.001 (ANOVA One-way Test).

To identify mechanisms how *BRD4i and CDK9i* synergistically induce apoptosis, the expression of anti-apoptotic genes was evaluated. Alteration of global gene transcription by CDK9i affects anti-apoptotic genes like MCL1 [33]. To assess if combined use of CDK9i and BRD4i synergistically inhibits transcription of anti-apoptotic genes, expression of *MCL1*, *BCL6* and *BTG1* was analyzed by real-time PCR. CDK9i by LDC067 or DRB as single compounds decreased expression of these genes, while BRD4i slightly increased or did not affect gene expression (Figure 2A and 2B). CDK9i plus BRD4i (JQ1 plus LDC067 or iBET plus DRB) synergistically down-regulates *MCL1, BCL6* and *BTG1* (Figure 2A and 2B).

### BRD4i and CDK9i synergistically down-regulate MYC

The inhibitory action of BRD4 on tumor cell proliferation has been attributed to inhibition of the *cMYC* oncogene [34, 35]. MYC is involved in the tumorigenesis of several cancer entities including RT [10]. MYC directly recruits P-TEFb to its target genes, where MYC-induced transcription mainly is promoted by CDK9 [36]. BRD4i down-regulates MYC and the expression of MYC target genes by preventing P-TEFb recruitment [32], while CDK9i directly impairs P-TEFb activity.

Given the reported role of MYC in RT, we analyzed the effect of combined BRD4i/CDK9i on *cMYC* expression in rhabdoid tumor cell lines. As expected, BRD4i led to a slight to moderate repression of MYC expression in multiple RT lines (Figure 2C-2E). CDK9i reduced MYC expression at moderate to high concentrations (LDC067: 5-10 μM or DRB: 12.5 μM – 50 μM) (Figure 2C and 2D). Strikingly, the combined application of BRD4i and CDK9i strongly reduced nascent *MYC* mRNA (Figure 2C and 2D) and MYC protein levels (Figure 2E) in a synergistic manner, even when used at low concentrations.

### BRD4i and CDK9i act synergistically on housekeeping genes

**Figure 3:**
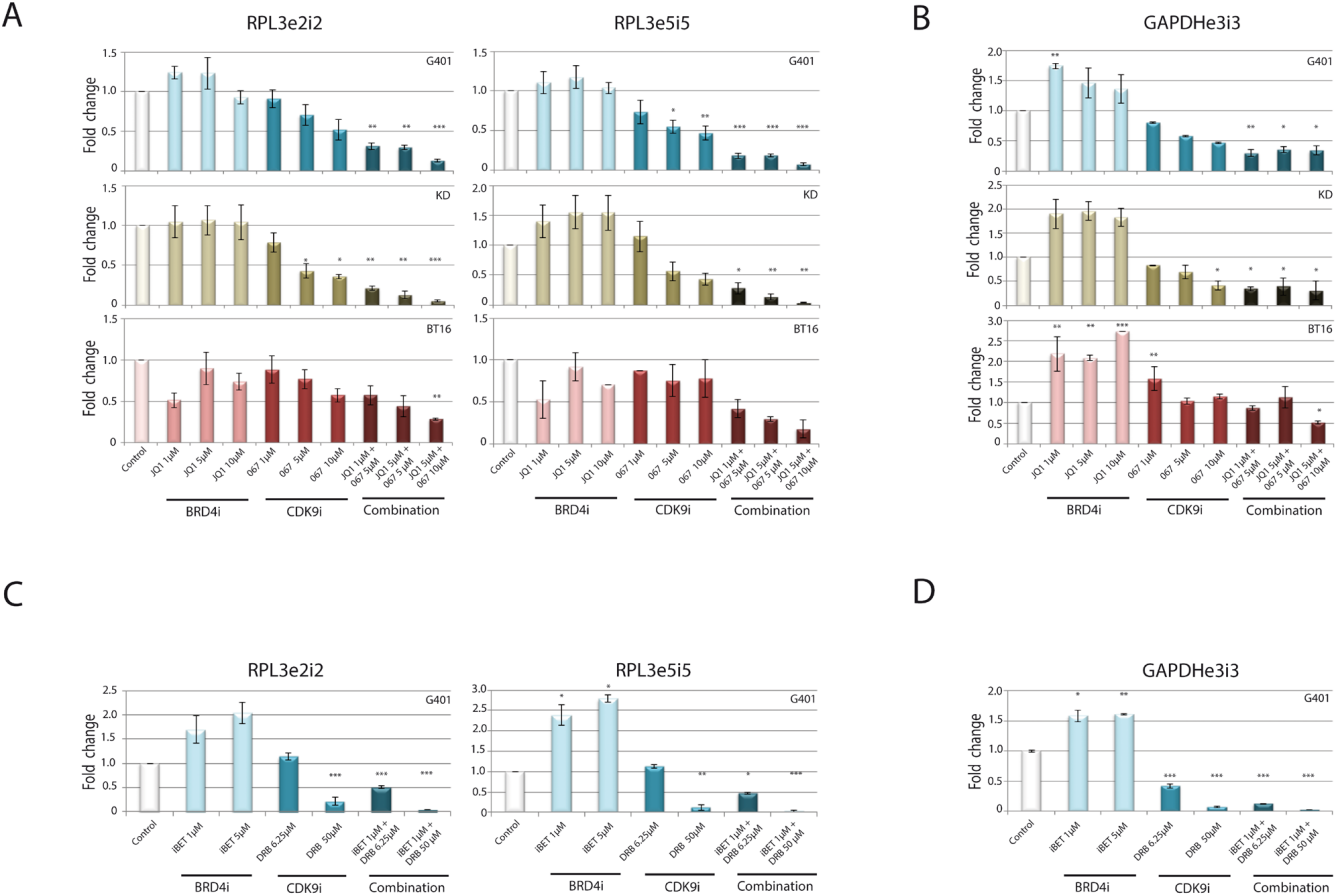
Combined BRD4 and CDK9 targeting acts synergistically on inhibition of general transcription To detect effects of combined treatment strategy on general transcription, the indicated RT cell lines were incubated for 120 min with different concentrations of JQ1/ LDC067 **(A, B)** or iBET/DRB **(C, D)** alone or in combination of both inhibitors. Gene expression was analyzed by RT-qPCR using different exon-intron and exon-exon primer pairs. Newly synthesized mRNA of the housekeeping genes *RPL3* **(A, C)** and *GAPDH* **(B, D)** is shown. Simultaneous application of the inhibitors significantly reduces the expression of these genes indicating a general blockade of the transcription process. *p<0.05, **p < 0.01, ***p < 0.001 (ANOVA One-way Test).

BRD4i and CDK9i synergistically impede transcription of anti-apoptotic genes as well as the oncogene *cMYC*. As BRD4 recruits P-TEFb [29], we hypothesized that inhibition of BRD4 and CDK9 synergistically impair the general transcription process. To test this hypothesis, we examined expression of nascent RNAs for housekeeping genes whose mRNA expression is normally unchanged. Primers designed to span exon-intron junctions revealed differences in *de novo* synthesized RNA of the *RPL3* and *GAPDH* genes. CDK9i alone diminished transcription of *RPL3* and *GAPDH* in a dose-dependent manner (Figure 3A-3D), while BRD4i induced transcription of *RPL3* and *GAPDH*, as previously observed [37].

Even at low doses, combined administration of both inhibitors heavily impaired transcription of both *RPL3* and *GAPDH* genes.

Overall, our data demonstrate that combined inhibition of both CDK9 and BRD4 significantly reduces transcription at important anti-proliferative-, anti-apoptotic genes and housekeeping genes, which might imply that impairment of gene expression occurs at a genome-wide level.

## DISCUSSION

Rhabdoid tumors are aggressive pediatric malignancies. Intensive multimodal therapeutic approaches, including chemotherapy, radiotherapy and surgery, regularly fail to cure this disease. Insights in tumor biology may contribute to acquire new molecular targets for chemotherapeutic efforts. Biallelic loss of *SMARCB1* results in deregulation of multiple pathways in RT [10]. Altered signaling cascades described so far involve deregulated transcription of target genes such as *MYC* and other cancer drivers [8-11]. In contrast to gain-of-function mutations or amplifications, it is not possible to directly target a loss of gene function. As an alternative approach we used specific inhibitors of BRD4 and CDK9 to target the deregulated transcription machinery in RT. Effects on blocking RT cell proliferation by BRD4i and CDK9i described in this manuscript are probably not tumor entity specific and might also be of interest for therapeutical approaches in other entities.

In recent years, several small molecule inhibitors have been reported to display significant antitumor activity. We focused on two groups of inhibitors: those targeting bromodomains and those targeting CDK9. Both classes of compounds repress proliferation, induce apoptosis, and reduce tumor growth, with many of these now employed in clinical trials [27, 28].

Although BRD4 and CDK9 are not overexpressed in RT when compared to normal tissue (Supplementary Figure 2) they function as key elements of the transcription machinery and epigenetic regulation, leading us to the hypothesis that they might have an impact on rapidly proliferating RT cells as well. Indeed, two novel BET-inhibiting agents, JQ1 and iBET, exhibited significant anti-proliferative effects in multiple RT cell lines. Treatment of RT cell lines with BET inhibitors caused a cell cycle arrest in G1, but was not (iBET) or modestly (JQ1) accompanied by apoptosis. In analogue experiments we evaluated the effects of specific CDK9i, DRB and LDC067 *in vitro*. These inhibitors showed anti-proliferative effects and triggered cell cycle arrest accompanied by apoptosis *in vitro*, going in line with increased p53 activity as reported by our group and others before [38, 39].

The well-studied functional interactions between BRD4 and CDK9 raised the idea of inhibiting both molecules simultaneously, aiming to achieve sustainable depletion of neoplastic activity by arresting tumoral transcription and induction of apoptosis. Combining both inhibitors triggered a significant down-regulation of anti-apoptotic genes and, finally, induced apoptosis even in low concentrations. Concomitant with that we observed a nearly complete abolishment of c-MYC protein expression. These results were confirmed by *in vivo* experiments, using a NOD/SCID xenograft-model, which demonstrated significant tumor growth suppression for mice receiving BRD4-inhibitor JQ1 and CDK9-inhibitor LDC067 at the same time.

Recently another group has found synergistic effects of BRD4i and CDK9i in other tumor entities as well. Lu et al. described HeLa- and NSCLS-cells undergoing CDK9-inhibition to react with a strong increase of MYC-activity that was achieved by amplified BRD4-mediated recruitment of P-TEFb to the MYC-locus. This was reflected by higher concentration of CDK9 and RNA Pol II at the MYC-locus and, consequently, increased transcriptional activity. Hence, simultaneous inhibition of CDK9 and BRD4 resulted in stop of proliferation and apoptosis [39], possibly mediated through altered levels of MYC-dependent regulators of apoptosis. Anyway, MYC seems to play a crucial role in understanding the mechanisms underlying our observations, as it plays a mediating role between BRD4 and P-TEFb and, of note, is frequently altered in RT [10, 40]. MYC recruits P-TEFb to its target genes where MYC-induced transcription mainly is conducted by CDK9 [36]. This effect might be prevented by inhibition of CDK9 [41]. However, regulation of MYC-activity depends on BET-proteins such as BRD4: Experiments with small molecular inhibitors prove the necessity of intact BRD4-function for MYC-triggered processes [32, 42, 43]. Like other oncogenes, MYC takes full effect through interaction with so-called super-enhancers, DNA-sections with multiple binding sites for transcription factors that have exceeding impact on transcriptional regulation [44]. Inhibition of BRD4 leads to loss of BRD4 and CDK9 at super-enhancers with depletion of newly synthesized DNA as a consequence, an effect that especially affects super-enhancers [34, 35, 45]. Inhibiting the MYC-interactors BRD4 and CDK9 at the same time point results in a notable decline of newly synthesized MYC-mRNA even at low concentrations. Of note, not only *MYC*, but also diverse anti-apoptotic genes, namely *BCL6, BTG, XIAP*, as well as the housekeeping gene *RPL3*, were affected in the same manner, what might indicate more general effects on transcription. Termination of transcription regulation leads to apoptosis. p53 is strongly increased following the inhibition of CDK9 by reduced expression of MDM2, an ubiquitin-ligase which prevents accumulation of p53 [46, 47]. Interestingly, this effect is augmented by simultaneous inhibition of BRD4. Regarding the interaction of BRD4 and CDK9 – BRD4 recruits P-TEF-b/CDK9 to the promotor and may additionally serve as an atypical CTD-kinase of RNA Pol II [22, 48] – one could hypothesize that the steps of molecular interactions which are necessary to reinitiate transcription are hit at two points of the same signaling way, leading to transcriptional stop and, finally, apoptosis. Another explanation for the observed effects might be the existence of two equivalent signaling cascades, with MYC as a potential substitute for inhibited CDK9, which then again would be affected through BRD4-inhibition via its sensitivity for disturbance of super-enhancer-function. Being observed in all available RT cell lines, these results indicate for a general mechanism that may be targetable in rhabdoid tumors of all anatomical origins with only slight variations regarding the extent of apoptosis induction, that could be due to individual expression of CDK9, BRD4 and, particularly, MYC. Interestingly, further entities seem to be sensitive to the combined BRD4i and CDK9i treatment, e. g. HeLa-, H1792 NSCLC cells [39] and several osteosarcoma cell lines [53]. The combined effects of BRD4 and CDK9 inhibition on blocking tumor cell proliferation might be independent of the MYC- expression, as H1792 is a MYC-amplified cell line and HeLa cells do not harbour any MYC alteration.

In this study, we demonstrate a promising therapeutical approach for highly aggressive RT through combined inhibition of BRD4 and CDK9. Further understanding of the complex interactions between CDK9, BRD4 and MYC could be a next step towards the establishment of new agents for innovative therapy options for this and other malignancies.

## MATERIALS AND METHODS

### Cell culture

The experiments were performed on different RT cell lines: G401 and MON (RTK), BT16 (AT/RT), KD and A204 (MRT). G401 and A204 cell lines were obtained from Michael Frühwald (University Children’s Hospital Muenster, Department of Pediatric Hematology and Oncology, Muenster, Germany/ Children’s Hospital Augsburg, Swabian Children’s Cancer Center, Augsburg, Germany). MON and KD cell lines were obtained from Sabine Schleicher (University Hospital Tübingen, Clinic for Pediatrics, Department I Hematology/Oncology, Tübingen, Germany). BT16 cell line was obtained from Martin Hasselblatt (Institute of Neuropathology, University Hospital Muenster, Muenster, Germany). G401, MON, KD and A204 cell lines were cultured in DMEM high glucose formulation (Invitrogen #11965-092, Karlsruhe, Germany) with 10% fetal bovine serum (PAA Cell Culture Company #A15-151, Cambridge, UK), 1% L-Glutamine (Gibco #25030-018, Life Technologies GmbH, Darmstadt, Deutschland) and 1% penicillin/streptomycin (Gibco #15140-122, Life Technologies). BT16 cells were cultured in DMEM high glucose formulation supplemented with 16% Fetal Bovine Serum (Gibco #10270-106, Life Technologies) and 1% penicillin/streptomycin. All cell lines were incubated at 37°C with 5% CO_2_. All lines have been authenticated using STR-PCR.

### Bromodomain inhibitors, Cdk9-inhibitors

iBET (I-BET762) (GSK525762A; Molecular formula: C_22_H_22_C_l_N_5_O_2_) was obtained from Cayman Chemical Company (Ann Arbor, MI, USA). JQ1 (Molecular formula: C_23_H_25_C_l_N_4_O_2_S) was synthesized by James Bradner’s lab (DFCI, Boston, USA).

DRB 5, 6-Dichlorobenzimidazole 1-β-D-ribofuranoside (Molecular formula: C_12_H_12_Cl_2_N_2_O_4_) was obtained from Sigma Aldrich (#D1916, Taufkirchen, Germany). LDC067 (3-((6-(2-methoxyphenyl) pyrimidin-4-yl) phenyl) methanesulfonamide (Molecular Formula: C_18_H_18_N_4_O_3_S) was synthesized by us. All compounds were dissolved in DMSO and stored at -20°C in the dark until use. For mouse experiments JQ1 and LDC067 were further diluted in sterile PBS.

### Cytotoxicity assay

Cytotoxicity assays were performed as described previously [49]. Cell suspensions (5000 cells/ 50 μl) of different RT cell lines, G401, MON, A204, KD and BT16 were seeded into 96-well-plates. Cells were allowed to reach exponential growth before 50 μl of cell culture medium containing the inhibitor drugs at different concentrations were added. Each drug concentration (0, 0.01, 0.1, 1, 10 and 100 μM) was tested in 3 biological replicates. For experiments with combined treatment, we used compound 1 in increasing concentrations as in single compound experiments (0, 0.01, 0.1, 1, 10 and 100 μM). Compound 2 was used at 1/10 dilution of the concentration of compound 1. After 48 h, cells were incubated for 4 h with MTT reagent according to the manufacturer's instructions (Cell Growth Kit, Millipore, Germany). Formazan crystals were dissolved in Isopropanol-0.04N HCl buffer. Cell density was determined by measuring absorbance spectrophotometrically at 570 nm and a reference of 650 nm, using a microplate photometer Multiskan Ascent multiplate reader (Labsystems, Helsinki, Finland). Experiments were performed three times.

### Analysis of combined drug effects on cytotoxicity

To evaluate drug combination effects we analyzed cytotoxicity assay data using the median effect method by Chou and Talalay [50]. The fraction of unaffected cells was defined as the proportion of living cells compared to the control. The combination index indicates synergism if CI < 1, antagonism for CI > 1 and an additive effect for CI ≈1. Values of the CI were determined at the IC_50_ concentration (fraction affected = 0.5). The method was implemented in the statistical software R (Version 2.15.1).

### Cell cycle and apoptosis analysis

The effects of BRD4 and CDK9 inhibitors on cell cycle and apoptosis were tested in diverse RT cells lines: G401, MON, BT16, KD, and A204. Cells were incubated with different concentrations of drugs as follows: JQ1 (1, 5, 10 μM), LDC067 (1, 5, 10 μM) or combination of JQ1 plus LDC067 (1 μM + 5 μM, 5 μM + 5 μM, 5 μM + 10 μM, respectively); iBET (1μM), DRB (6.25, 12.5, 25, 50 μM) or combination of iBET plus DRB (1 μM + 6.25 μM, 1 μM + 12.5 μM, 1 μM + 25 μM, 1 μM + 50 μM respectively) for 48h before cells were harvested and washed with PBS. For cell cycle, 100 μl of cell suspension were incubated with 4', 6-diamidino-2-phenylindole (DAPI) and measured using CyFlow space flow cytometry system (Partec, Muenster, Germany). Data from cell cycle analysis were analyzed using FlowJo (Tree Star Inc., Ashland, OR, USA). The calculation of the area under the curve during cell cycle analysis was achieved using the Watson- or Dean-Jett-Fox-models on all samples of a particular cell line. Induction of apoptosis following single or combined treatment with these compounds was detected by staining the cells with Annexin-FITC and Propidium Iodide (BD Biosciences, Heidelberg, Germany) and measured with CyFlow space flow cytometry system (Partec, Muenster, Germany). Data were analyzed using FlowJo (Tree Star Inc., Ashland, OR, USA).

### Immunoblotting

Cultured cells were harvested in icecold PBS and lysed in Lämmli buffer followed by sonification (30’’ On-OFF cycle, 15 minutes). Thirty to 50 μg of total protein was loaded into 10% or 12% SDS-PAGE gels and electrophoretically transferred onto polyvinylidene difluoride membrane (PVDF Bio-Rad Laboratories GmbH, Munich, Germany) by using a semidry blotting system (Trans-Blot^®^Turbo blotting System, Bio-Rad). After that, the membrane was blocked in 5% (w/v) defatted powdered milk/Tris–buffered saline 0.1% Tween 20 for 1, 5 h at room temperature and incubated overnight with the desired primary antibodies at 4°C. After rinse, the membrane was incubated for 1 h at RT with the corresponding secondary antibodies conjugated with horseradish peroxidase (Santa Cruz Biotechnology). Finally, protein visualization was performed using Western Lightning Plus ECL (Perkin Elmer Inc., Waltham, MA, USA) chemiluminescent reagent.

The following primary and secondary antibodies were used: c-MYC [9E10] Chip Grade (ab32) (Abcam, Cambridge, UK), and α-Tubulin (sc 23948), (Santa Cruz Biotechnology, CA, USA), Goat Anti-Rabbit IgG (H+L) (# 111-035-045) (Jackson ImmunoResearch, Suffolk, UK); Goat Anti-Mouse IgG + IgM (H+L) (#115-035-044) (Jackson ImmunoResearch, Suffolk, UK).

### Real time qPCR analysis

Real-time qPCR analysis was performed according to a previously published protocol [51]. Total RNA was isolated using TRIzol^®^ reagent (Invitrogen Life Technologies GmbH, Darmstadt, Germany). cDNA was generated using Prime Script RT reagent Kit (Takara Bio Inc.; Japan) and applied according to the manufacturer's protocol. Real-time quantitative PCR (qRT-PCR) was performed using SYBR Green (Promega; Germany) in the Step One Plus detection system (Applied Biosystems; Germany). For each primer set, post-amplification melting curves were analyzed to verify the specific amplification of the expected PCR product. Gene expression was determined by using the comparative cycle threshold (ΔΔCt) method. All assays were performed in triplicates.

The following primers were used:

RPL3-e9f: CCTTAAGTTCATTGACACCACCT; RPL3 e10r: CTCACCATGAATGCTTTCTTCT
RPL3 e2f: GATACAAGGCTGGCATGACTC; RPL3 i2r: CCCCAACTTTTAGGATGTCTGTA
RPL3 e5f: CAAGGGCAAAGGCTACAAAG; RPL3 i5r: GAATGGTTCTACACTGTCCGATT
MYC e2f: TCAAGAGGTGCCACGTCTCC; MYC e3r: TCTTGGCAGCAGGATAGTCCTT
MYC i1f: TGCTAAAGGAGTGATTTCTATTTCC; MYC i1r: AGGTGATCCAGACTCTGACCTT
GAPDH e2f: GAAGGTGAAGGTCGGAGTC; GAPDH e4r: GAAGATGGTGATGGGATTTC
GAPDH e3f: AATGACCCCTTCATTGACCTC ; GAPDH i3r: GGGGGAATACGTGAGGGTAT
BCL6 f: ACACATCTCGGCTCAATTTGC ; BCL6 r: AGTGTCCACAACATGCTCCAT
BTG1 f: TCCTCTGATTGGACAGGCAG ; BTG1 r: GCTGTTTTGAGTGCTACCTCCT
MCL1 f: TGCTTCGGAAACTGGACATCA; MCL1 r: TAGCCACAAAGGCACCAAAAG

### *In vivo* rhabdoid tumor xenograft experiments

*In vivo* experiments were performed as described previously [52]. Briefly, G401 cells were used to generate the xenograft mouse model. 8-week-old Non-obese-diabetic–severe combined immunodeficient mice, NOD/SCID (NOD.CB17-*Prkdc*^*scid*^/J, Jackson Laboratory) were irradiated with a single dose of 3.5 Gy from a linear accelerator 1 day before transplantation. Then, 1 x 10^7^ cells were injected in the flank of the animals. Tumor growth was monitored measuring the two-dimensional tumor size with a digital caliper. Tumor volume was calculated after the equation: (a x b^2^)/2.

When tumor volumes reached 50-80 mm^3^, mice were randomized into control and treatment groups: JQ1, LDC067 and JQ1 + LDC067 (n= 6 for each condition). Mice were treated intraperitoneally twice a week during 3 weeks and afterwards observed for 3 more weeks. The administration of compounds was as follows: JQ1: 30 mg/kg/day, LDC067: 10 mg/kg/day. JQ1+ LDC067: JQ1 (30 mg/kg/day) + LDC067 (10 mg/kg/day).

All experimental manipulations were performed under sterile conditions in a laminar flow chamber. All animal experiments were undertaken in accordance to the guidelines provided by the local regulatory authorities.

### Statistical analyses

All Data are represented as mean values ± SEM. For comparison of more than 2 values ANOVA One-way test with Turkeys post-test was used. All statistical analyses were performed using Graph Pad Prism 6.0 software. Significance was assumed when p< 0.05.

## SUPPLEMENTARY MATERIALS FIGURES



## Abbreviations

RT: rhabdoid tumors
RNAPII: RNA polymerase II
RTK: rhabdoid tumors of the kidney
AT/RT: atypical teratoid/rhabdoid tumor
MRT: malignant rhabdoid tumors
NOD/SCID: non-obese-diabetic–severe combined immunodeficient mice
CDK: cyclin dependent kinase
CDK9i: CDK9 inhibitor
BRD4i: BRD4 inhibitor
