# Supplementary material for: Combined BRD4 and CDK9 inhibition as a new therapeutic approach in malignant rhabdoid tumors
Source: Oncotarget. 2017 Jun 21;8(49):84986–95. doi: 10.18632/oncotarget.18583 (PMC5689588; doi:10.18632/oncotarget.18583)
Supplement: Supplementary file 1 [file oncotarget-08-84986-s001.pdf]

Combined BRD4 and CDK9 inhibition as a new therapeutic approach in malignant rhabdoid tumors

SUPPLEMENTARY FIGURES

A

Cell line	Treatment	IC_{50}(μM)	R^2	CI
G401	JQ1	42.17	0.96	
	067	158.45	0.93	
	Combination	149.97	0.96	1.005
BT16	JQ1	23.43	0.99	
	067	39.28	0.99	
	Combination	7.83	0.97	0.217
KD	JQ1	42.67	0,97	
	067	142.6	0.99	
	Combination	103.83	0.97	1.005
A204	JQ1	30.36	0.93	
	067	52.19	0.95	
	Combination	23.84	0.93	0.771
MON	JQ1	40.98	0.99	
	067	92.52	0.94	
	Combination	23.64	0.93	0.56

C

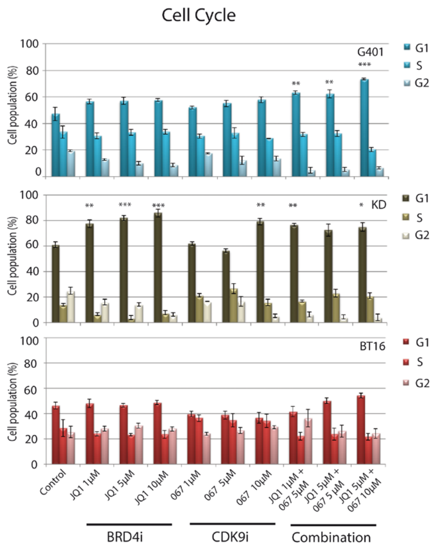

B

Cell line	Treatment	IC_{50}(μM)	R^2	CI
G401	iBET	23.99	0.84	
	DRB	376.99	0.71	
	Combination	8.95	0.82	0.05
BT16	iBET	189.4	0.98	
	DRB	65.21	0.99	
	Combination	56.64	0.91	0.373
KD	iBET	4.51	0.91	
	DRB	48.59	0.99	
	Combination	3.12	0.94	0.638
A204	iBET	27.07	0.99	
	DRB	142.85	0.97	
	Combination	10.6	0.93	0.365
MON	iBET	5.08	0.99	
	DRB	73.04	0.94	
	Combination	3.7	0.94	0.671

D

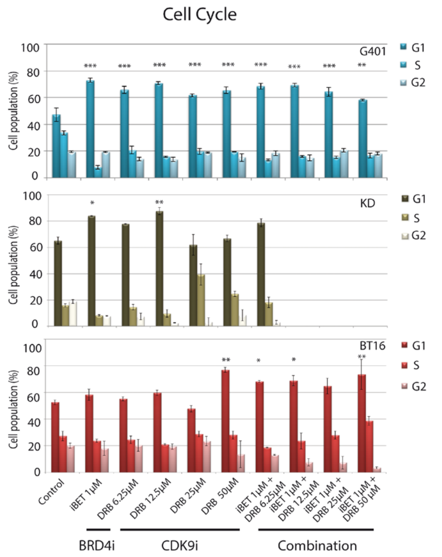

Supplementary Figure 1: Combined inhibition of BRD4 and CDK9 synergistically inhibits cell proliferation and induces cell cycle arrest in rhabdoid tumor cells *in vitro*. Different RT cell lines were incubated with BRD4 and CDK9 inhibitors at a range of concentrations (0 – 100 μM) as single compounds or as a combination with JQ1/ LDC067 **(A)** or iBET/DRB **(B)**. IC_{50} and CI values obtained by MTT assays are shown. Combined indexes (CI) were calculated using Chou-Thalalay's algorithm. CI indicates cooperation (synergistic or additive) between BRD4 and CDK9 inhibitors after simultaneous treatments. Cell cycle profile analyzed by flow cytometry after treatment with BRD4 and CDK9 inhibitors alone or together in the indicated cell lines is shown. Simultaneous application of JQ1/ LDC067 **(C)** or iBET/DRB **(D)** elicits a G1 Phase arrest. IC_{50}: Drug concentrations causing 50% growth inhibition, CI: Combination Index: indicates synergism if CI < 1, antagonism for CI > 1 and an additive effect for CI ≈1. *p<0.05, **p < 0.01, ***p < 0.001 (ANOVA One-way Test).

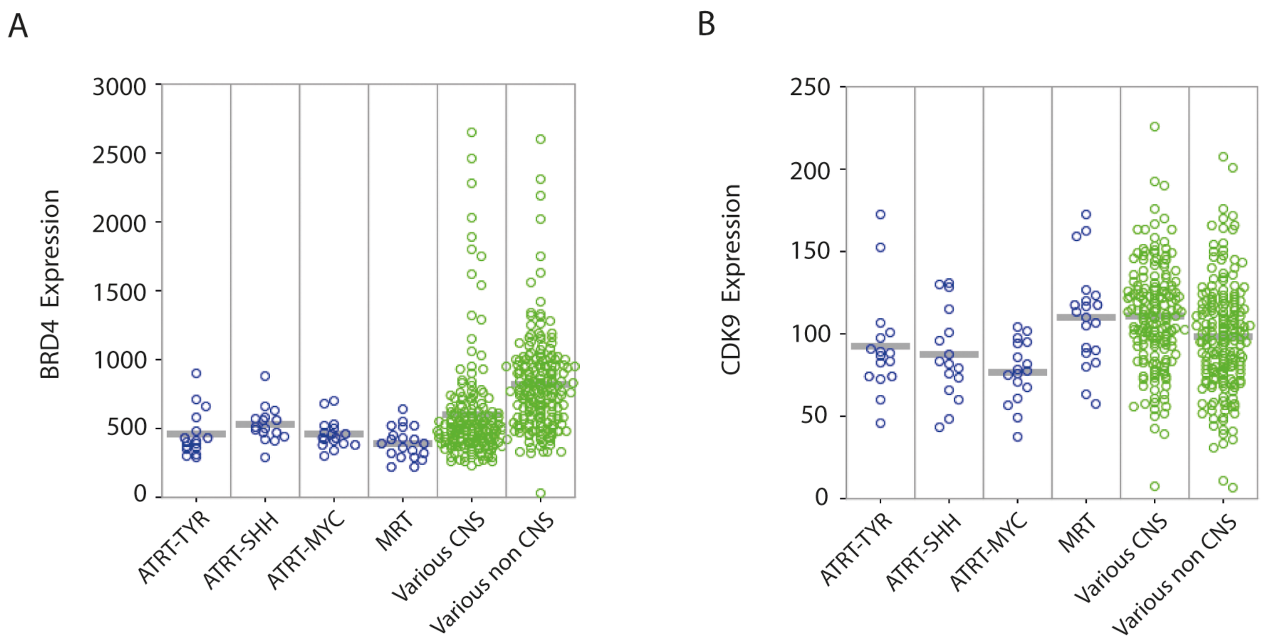

Supplementary Figure 2: Expression of BRD4 and CDK9 in rhabdoid tumors. Gene expression profiling analysis of BRD4 **(A)** and CDK9 **(B)** performed on human samples of intracranial (AT/RT) and extracranial (MRT) tumors show a similar expression of both genes in tumors compared to healthy tissue.
